# Purification, Conformational Analysis and Cytotoxic Activities of Host-Defense Peptides from the Giant Gladiator Treefrog *Boana boans* (Hylidae: Hylinae)

**DOI:** 10.3390/antibiotics12071102

**Published:** 2023-06-25

**Authors:** J. Michael Conlon, Laure Guilhaudis, Samir Attoub, Laurent Coquet, Jérôme Leprince, Thierry Jouenne, Milena Mechkarska

**Affiliations:** 1 Diabetes Research Centre, School of Biomedical Sciences, University of Ulster, Coleraine BT52 1SA, UK; 2 Laboratoire COBRA (UMR 6014 & FR 3038), UNIROUEN, INSA de Rouen, CNRS, Université Rouen Normandie, 76000 Rouen, France; laure.guilhaudis@univ-rouen.fr; 3 Department of Pharmacology and Therapeutics, College of Medicine and Health Sciences, United Arab Emirates University, Al Ain 17666, United Arab Emirates; samir.attoub@uaeu.ac.ae; 4 CNRS UAR2026 HeRacLeS-PISSARO, CNRS UMR 6270 PBS, Université Rouen Normandie, 76821 Mont-Saint-Aignan, France; laurent.coquet@univ-rouen.fr (L.C.); thierry.jouenne@univ-rouen.fr (T.J.); 5 Inserm U1239, PRIMACEN, Institute for Research and Innovation in Biomedicine (IRIB), Université Rouen Normandie, 76000 Rouen, France; jerome.leprince@univ-rouen.fr; 6 Department of Life Sciences, Faculty of Science and Technology, St. Augustine Campus, The University of The West Indies, St. Augustine, Trinidad and Tobago; milena.mechkarska@sta.uwi.edu

**Keywords:** antimicrobial peptide, anti-cancer, frog skin, circular dichroism, Hylidae, *Boana*

## Abstract

Frogs from the extensive amphibian family Hylidae are a rich source of peptides with therapeutic potential. Peptidomic analysis of norepinephrine-stimulated skin secretions from the Giant Gladiator Treefrog *Boana boans* (Hylidae: Hylinae) collected in Trinidad led to the isolation and structural characterization of five host-defense peptides with limited structural similarity to figainin 2 and picturin peptides from other frog species belonging to the genus *Boana*. In addition, the skin secretions contained high concentrations of tryptophyllin-BN (WRPFPFL) in both C-terminally α-amidated and non-amidated forms. Figainin 2BN (FLGVALKLGKVLG KALLPLASSLLHSQ) and picturin 1BN (GIFKDTLKKVVAAVLTTVADNIHPK) adopt α-helical conformations in trifluroethanol–water mixtures and in the presence of cell membrane models (sodium dodecylsulfate and dodecylphosphocholine micelles). The CD data also indicate contributions from turn structures. Both peptides and picturin 2BN (GLMDMLKKVGKVALT VAKSALLP) inhibited the growth of clinically relevant Gram-negative and Gram-positive bacteria with MIC values in the range 7.8–62.5 µM. Figainin 2BN was potently cytotoxic to A549, MDA-MB-231 and HT-29 human tumor-derived cells (LC_50_ = 7–14 µM) but displayed comparable potency against non-neoplastic HUVEC cells (LC_50_ = 15 µM) indicative of lack of selectivity for cancer cells.

## 1. Introduction

The very extensive amphibian family Hylidae, currently comprising 1044 recognized species, is divided into three sub-families: Hylinae (746 species), Pelodryadinae (231 species) and Phyllomedusinae (67 species) [1]. Skin secretions from Australian treefrogs belonging to the genus *Litoria* (Pelodryadinae) [2] and from South American frogs from the genus *Phyllomedusa* (Phyllomedusinae) [3] have proved to be a rich storehouse of biologically active peptides whose properties include myotropic, insulinotropic, immunomodulatory and anti-tumor actions as well as host-defense peptides (HDPs) with antimicrobial actions against bacteria, parasites, protozoa and viruses. Frogs from the diverse sub-family Hylinae are found in North America, southward throughout Mexico and Central America, and also throughout much of temperate Eurasia, Japan and extreme northern Africa [1]. Within Hylinae, the genus *Boana*, currently comprising 99 species, has been the most extensively investigated for the presence of HDPs in skin secretions. Previous studies have identified families of structurally related peptides with varying degrees of antimicrobial activity including hylins from *Boana albopunctata* [4], *Boana lundii* [5], *Boana prasina* [6] and *Boana pulchella* [7]; raniseptins [8], figainin 1 [9] and figainin 2 [10] from *Boana raniceps*; and picturins and pictuseptins from *Boana picturata* [11].

The Giant Gladiator Treefrog *Boana boans* (also known as the Rusty Treefrog) was formerly classified as *Hyla boans* and subsequently as *Hypsiboas boans* [1]. The mature frog is large (males 101–128 mm, females 91–123 mm snout-to-vent length), arboreal and nocturnal. Its trivial name is derived from the observation that males use exposed bones on their thumbs to fight in defense of egg-laying sites. The species is widely distributed in the Amazon Basin of Bolivia, Brazil, Peru, Ecuador and Colombia as well as Guyana, Panama and Venezuela [1,12]. An account of the populations on Trinidad and Tobago has been provided by Murphy et al. [13]. The population is stable and *B. boans* is currently categorized in the IUCN Global Red List as a Species of Least Concern [14].

This study describes the purification and structural characterization of HDPs from norepinephrine-stimulated skin secretions from *B. boans* frogs collected in Trinidad. Conformational analysis of the peptides in a range of membrane-mimetic media and an investigation of the cytotoxic activities against Gram-negative and Gram-positive bacteria and a range of human cell lines are presented. On the basis of limited structural similarity to previously described frog skin HDPs, these peptides have been assigned to the figainin 2 [10] and picturin peptide [11] groups. In addition, small tryptophan- and proline-containing peptides tentatively assigned to the tryptophyllin group of frog skin peptides [15] were isolated in high yield.

## 2. Results

### 2.1. Purification of the Peptides

**Figure 1:**
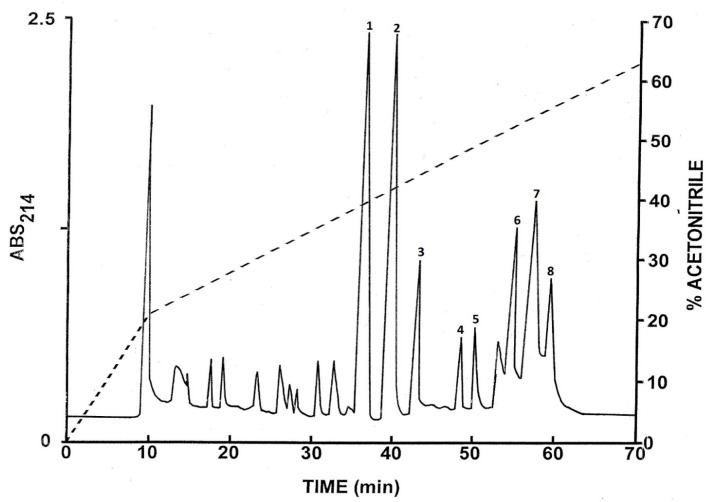
Reversed-phase HPLC on a semipreparative Vydac C-18 column of pooled skin secretions from *B. boans* collected in Trinidad after partial purification on Sep-Pak C-18 cartridges. The dashed line shows the concentration of acetonitrile in the eluting solvent. The peaks denoted 1–8 contained peptides that were purified further.

**Figure 2:**
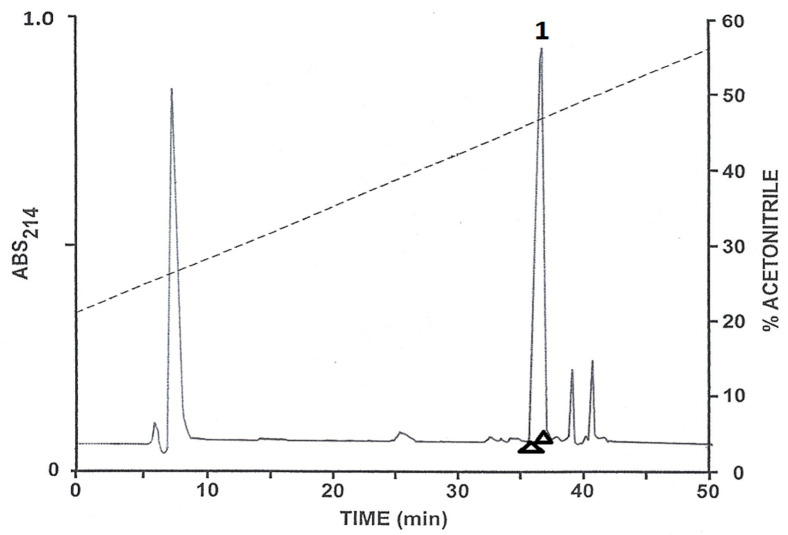
Reversed-phase HPLC on a semipreparative Vydac C-4 column of tryptophyllin-BN (peak 1) from *B. boans* after partial purification on a Vydac C-18 column. The arrowheads show where peak collection began and ended. The dashed line shows the concentration of acetonitrile in the eluting solvent.

The pooled skin secretions, after partial purification on Sep-Pak C-18 cartridges, were chromatographed on a semipreparative Vydac C-18 reversed-phase HPLC column (Figure 1). The prominent peaks designated 1–8 were collected and subjected to further purification. Subsequent structural analysis showed that peak 1 contained the C-terminally α-amidated form of tryptophyllin BN; peak 2, the non-amidated form of tryptophyllin BN; peak 4, figainin 2BN; peak 5, [Q27E]figainin 2BN; peak 6, picturin 2BN; peak 7, picturin 1BN; and peak 8, [N21D]picturin 1BN. Peak 3 contained a peptide that may comprise a post-translationally modified form of a tryptophyllin whose precise structure is currently under investigation. All peptides were purified to near homogeneity (purity > 98%) by means of further chromatography on a semipreparative Vydac C-4 column and, when necessary, on a Vydac C-8 column. The methodology is illustrated via the separation of tryptophyllin-BN from minor components on a Vydac C-4 column (Figure 2). The criteria used to assess purity were (1) symmetrical peak shape during final HPLC, (2) a single amino acid phenylthiohydantoin derivative detected during each cycle of Edman degradation and (3) a single component detected during mass spectrometry.

### 2.2. Structural Characterization

The primary structures of tryptophyllin BN, figainin 2BN, picturin 1BN and picturin 2BN were established without ambiguity via automated Edman degradation, and their complete primary structures are shown in Table 1.

The data indicated that tryptophyllin BN was also obtained in a non-C-terminally amidated form. Figainin 2BN was also isolated in a molecular form in which a single glutamine residue was replaced by glutamic acid, and picturin 1BN was isolated in a form in which a single asparagine residue was replaced by aspartic acid. The modified peptides were obtained in high yield, so they are more likely to be the products of separate genes rather than artefactually generated hydrolysis products produced during the extraction and purification procedures. The molecular masses of the peptides, determined using MALDI-TOF mass spectrometry, were consistent with the proposed structures and are also shown in Table 1. The calculated physicochemical properties of the five HDPs are presented in Table 2.

### 2.3. Conformational Analysis

**Figure 3:**
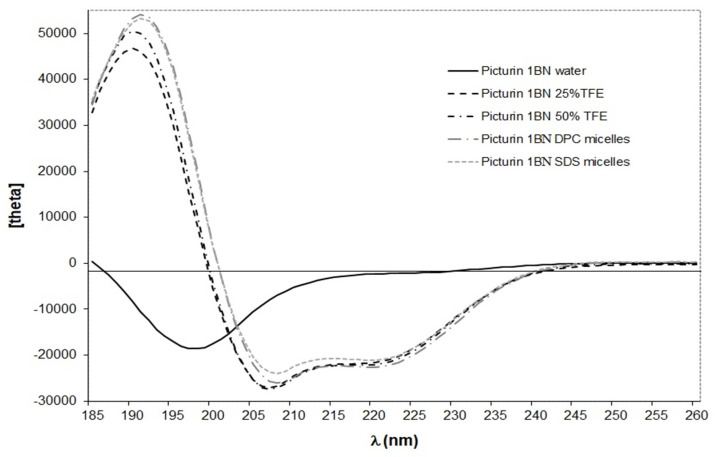
Circular dichroism spectra of figainin 2BN at room temperature and at a concentration of 0.25 mg·mL^−1^ in water (black solid line), 25% (*v*/*v*) TFE–water (black dashed line), 50% (*v*/*v*) TFE–water (black dashed-dotted line), 20 mM dodecylphosphocholine (DPC) aqueous solution (grey dash-dotted line) and 10 mM sodium dodecyl sulfate (SDS) aqueous solution (grey dashed line).

**Figure 4:**
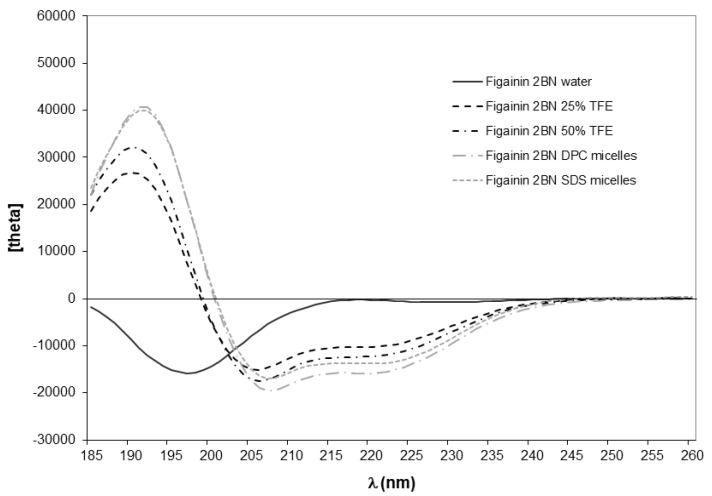
Circular dichroism spectra of picturin 1BN at room temperature and at a concentration of 0.25 mg·mL^−1^ in water (black solid line), 25% (*v*/*v*) TFE–water (black dashed line), 50% (*v*/*v*) TFE–water (black dashed-dotted line), 20 mM dodecylphosphocholine (DPC) aqueous solution (grey dash-dotted line) and 10 mM sodium dodecyl sulfate (SDS) aqueous solution (grey dashed line).

In common with the vast majority of frog skin HDPs studied to date [20,21], the CD spectra of synthetic replicates of figainin 2BN and picturin 1BN in water are characteristic of a predominantly random coil conformation, as evidenced by the negative band around 195 nm (Figure 3 and Figure 4). Peptide secondary structure predictions using the PED2D program [19] indicate that both peptides have a strong propensity for adopting extended α-helical conformations in an appropriate environment (Table 2). This prediction is consistent with circular dichroism (CD) analysis performed in the secondary-structure-inducing solvent trifluoroethanol (TFE) and in two membrane-mimetic environments, anionic sodium dodecyl sulfate (SDS) micelles and zwitterionic dodecylphosphocholine (DPC) micelles (Figure 3 and Figure 4). In these three media, CD spectra of both peptides exhibit two negative minima around 208 and 222 nm and a positive maximum near 192 nm, indicating the presence of an α-helical structure. Picturin 1BN displays very similar CD spectra between 200 and 245 nm in all media suggesting that, once adopted, the helical conformation is stable (Figure 4). In contrast, figainin 2BN presents increased dichroic signals at 208 and 222 nm with increasing TFE content and in the presence of micelles (Figure 3) showing the helical conformation is more stable in 50% TFE–water than in 25% TFE–water and is also stabilized through interaction with detergent micelles. In addition, higher mean residue molar ellipticities values are observed for picturin 1BN, indicating that it is more structured than figainin 2BN.

For both peptides, the most noticeable change observed in the CD spectra is for the maximum around 190 nm, whose intensity increases with the percentage of TFE and in the presence of micelles. The wavelengths of the positive and negative peaks are shifted slightly from the usual values associated with α-helical values (190 nm in TFE and 191 nm in micelles compared with the usual 192 nm, and 206 nm in TFE and 207 micelles compared with the usual 208 nm). This may be due to the presence of small amounts of additional secondary structural elements such as turns. The variable nature of turns in polypeptides makes analysis of their CD spectra difficult so that the impact of their presence on the overall CD spectrum of the peptide, which represents the combination of the individual spectra of each secondary structure found in the molecule, is difficult to assess.

Table 3 reports the percentage of secondary structure content estimated using the Dichroweb server [22,23,24] and the helical content calculated from the mean residue ellipticity at 222 nm using the Forood formula [25].

Although different algorithms give comparable estimates of the percentage of helix, the values determined with Dichroweb are generally higher. These differences are probably related to an underestimation of the ellipticities value at 222 nm due to the presence of turn structures. This hypothesis is in agreement with the presence of turns (up to 20%) calculated via the Dichroweb server for both peptides (Table 3). In most structure-inducing media (50% TFE, SDS micelles and DPC micelles), figainin 2BN exhibits between 40 and 49% of a helical structure, which corresponds to 11 to 13 residues out of 27. For picturin 1BN, the % helical domain comprises between 64 and 69%, corresponding to approximately 16 to 17 residues out of 25.

### 2.4. Antimicrobial Activities

Synthetic replicates of figainin 2BN, picturin 1BN and picturin 2B inhibited growth of reference strains of the Gram-negative bacteria *Escherichia coli*, *Klebsiella pneumoniae* and *Pseudomonas aeruginosa* and were active against an ampicillin-resistant strain of the Gram-positive bacterium *Staphylococcus aureus*. Minimum inhibitory concentrations (MICs) are shown in Table 4. In view of the fact that figainin 2BN showed high potency against *S. aureus*, its activities against Gram-positive *Enterococcus faecalis* and an antibiotic-resistant strain of *Enterococcus faecium* were also determined. The peptide showed moderately high potency against *E. faecium* but lower potency against *E. faecalis*.

The corresponding MIC value for ampicillin was 2.5 µg·mL^−1^ (*E. coli*), 2.5 µg·mL^−1^ for vancomycin (*S. aureus*), <3.1 µg·mL^−1^ for ampicillin (*E. faecalis*) and <0.19 µg·mL^−1^ for ciprofloxacin (*K. pneumoniae* and *P. aeruginosa*). *E. faecium* was resistant to both ampicillin and vancomycin (MICs > 100 µg·mL^−1^).

### 2.5. Cytotoxic Activities

Incubation of synthetic replicates of figainin 2BN, picturin 1BN and picturin 2BN for 24 h with human non-small cell lung adenocarcinoma A549 cells, human breast adenocarcinoma MDA-MB-231 cells, human colorectal adenocarcinoma HT-29 cells and human umbilical vein endothelial cells (HUVECs) resulted in a concentration-dependent decrease of cell viability determined via measurement of ATP concentrations. Table 5 shows the potencies of the peptides (LC_50_ values) revealing that figainin 2BN exhibited the greatest cytotoxic activity in all cases.

## 3. Discussion

**Figure 5:**
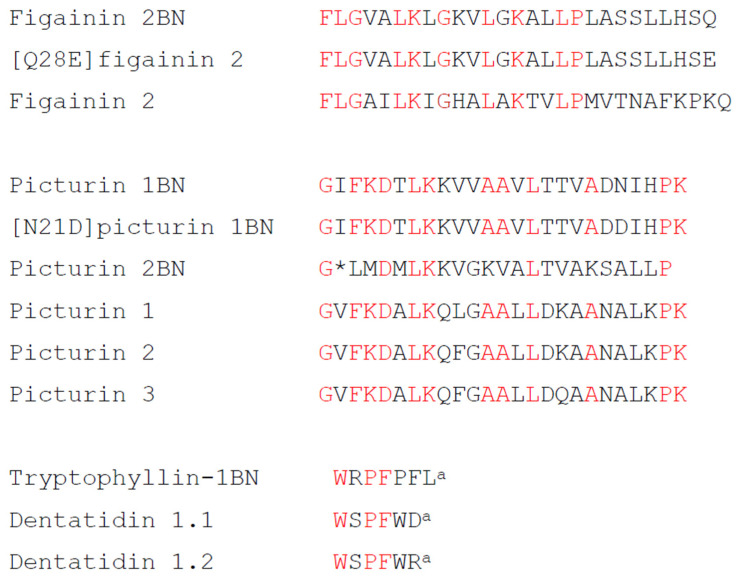
A comparison of the primary structure of figainin 2BN peptides from *B. boans* with the corresponding peptide from *B. raniceps* and the primary structures of picturin 1BN and picturin 2BN peptides from *B. boans* with the corresponding peptides from *B. picturata*. The primary structure of tryptophylin BN from *B. boans* is compared with those of similar peptides from *Litoria dentata*. Strongly conserved residues are shown in red. ^a^ denotes C-terminal α-amidation. A gap denoted by * is included to maximize structural similarity.

The study has identified five HDPs in norepinephrine-stimulated skin secretions from *B. boans* whose physicochemical characteristics are summarized in Table 2. The peptides were assigned to the figainin 2 and picturin peptide families on the basis of limited structural similarity with peptides from *B. raniceps* (figainin 2BN and [Q28E]figainin 2) [10] and *B. picturata* (picturin 1BN, [N21D]picturin 1BN and picturin 2BN) [11] (Figure 5).

HDPs isolated from frog skin secretions, including those from frogs belonging to the family Hylidae [26], have long been recognized as having therapeutic potential for development into agents for the treatment of infections produced by microorganisms that have developed resistance to conventional antibiotics. As shown in Table 4, synthetic replicates of figainin 2BN, picturin 1BN and picturin 2BN showed broad-spectrum antimicrobial activity, inhibiting the growth of both clinically relevant Gram-negative and Gram-positive bacteria. The focus of this article is on the structural aspects of antimicrobial peptides consistent with the theme of the Special Issue, and future studies will explore in a more comprehensive fashion the anti-bacterial and anti-fungal properties of the three peptides and the design of non-toxic analogs with increased potency.

**Figure 6:**
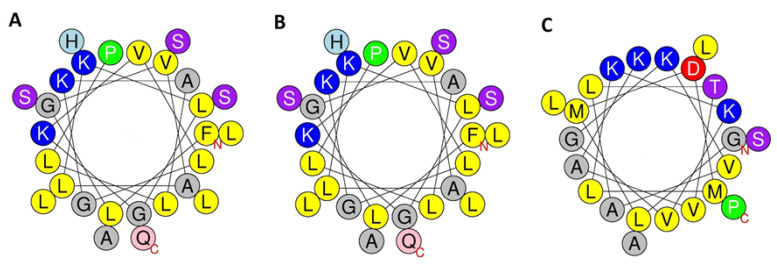
A Schiffer–Edmundson wheel representation of the conformation of (**A**) figainin 2BN, (**B**) picturin 1BN and (**C**) picturin 2BN. Basic amino acids are shown in blue, acidic amino acids are shown in red and strongly hydrophobic amino acids are shown in yellow.

With very few exceptions, frog skin HDPs are cationic, generally with a molecular charge at pH 7 between +1 and +5, contain a high proportion of hydrophobic residues and have the propensity to adopt an amphipathic α-helical conformation in a membrane-mimetic environment [20,21]. As shown in Table 2, the figainin 2BN and picturin peptides fulfill the criteria of cationicity and relatively high hydrophobicity, and CD studies have confirmed that the peptides adopt extended α-helical conformations in the presence of TFE and membrane-mimetic micelles. Schiffer–Edmundson wheel representations of the predicted helical regions of the peptides constructed using the HeliQuest web-server [18] demonstrate the amphipathic nature of the helices, consistent with their relatively high calculated hydrophobic moment (Figure 6). Each peptide is associated with an extended hydrophobic domain, comprising multiple Leu and Val residues on one face of the helix, that enables binding to (phospho)lipids in the bacterial cell membrane and a hydrophilic domain on the opposite face, containing multiple Lys residues, that promotes loss of integrity of the membrane [27].

The ability of figainin 2BN to produce death of non-small cell lung adenocarcinoma A549 cells, breast adenocarcinoma MDA-MB-231 cells and colorectal adenocarcinoma HT-29 cells during a 24 h incubation at relatively low concentrations (LC_50_ values in the range 7 to 14 µM) suggests that the peptide may represent a template for the design of anti-cancer drugs, particularly when the tumor is not responsive to conventional pharmaceutical agents [28]. Previous studies from the laboratory have shown that loss of cell viability produced by cytotoxic frog skin peptides under the conditions of the present study is rapid [29], suggesting that the mechanism of action involves non-specific destruction of the integrity of the plasma membrane rather than induction of apoptosis. Picturin 1BN and picturin 2BN were also cytotoxic to these cells, but their potencies were appreciably less (Table 5). However, it is important to note that the potential of figainin 2BN as an anti-cancer agent is limited by the fact that it shows little selectivity for cancer cells. The LC_50_ value against non-neoplastic human umbilical vein endothelial cells was 15 µM (Table 5).

The term tryptophyllin refers to a heterogeneous group of small (<10 amino acid residues) peptides containing one or more tryptophan and one or more proline residues. Multiple tryptophyllins have been identified in the skins of several species of frogs belonging to the sub-families Pelodryadinae and Phyllomedusinae (reviewed in [2,20,30]) but have also been isolated from skin secretions of the most primitive extant anuran, the tailed frog *Ascaphus truei* [31] and, more recently, from the venom of the snake *Bothrops moojeni* [32]. Classification of tryptophyllins together in a single group is largely a matter of convenience and does not necessarily mean that its members are evolutionarily or biosynthetically related. Tryptophyllin peptides with an N-terminal Trp residue such as the tryptophyllin 1BN isolated in this study are rare, and a comparison of the primary structure of the peptide with those of tryptophyllins identified to date indicates the greatest structural similarity with dentatidin 1.1 and 1.2 from the Australian treefrog *Litoria dentata* (Pelodryadinae) [33] (Figure 5). While individual tryptophyllins have shown a range of biological properties including myotropic activity [34], inhibition of neuronal nitric oxide synthesis [30], anti-proliferative activity against prostate cancer cell lines [34] and activity at opioid receptors [33], it is generally believed that their most physiologically relevant activity is their anti-oxidant and radical-scavenging properties, which protect the frog from ultraviolet- and radiation-induced damage in the environment [35].

## 4. Materials and Methods

### 4.1. Collection of Skin Secretions

Relevant permits approving the collection and sampling of live animals were granted by the Wildlife Section, Forestry Division, Trinidad and by the University of the West Indies (UWI) Campus Ethics Committee (CEC234/07/16). All experiments were carried out by authorized investigators. Two adult *B. boans* frogs (mean snout-to-vent length 90 mm, mean body weight 34.9 g, sex not determined) were collected at Santa Cruz, La Pastora settlement, Trinidad (GPS coordinates 10°43′56.9″ N 61°28′10.6″ W) in October 2021. The animals were sampled in the field and subsequently released unharmed at the site of capture. Each frog was injected via the dorsal lymph sac with norepinephrine hydrochloride (40 nmol/g body weight) and placed in distilled water (100 mL) for 15 min. The collection solution was acidified through the addition of concentrated hydrochloric acid (0.5 mL) and immediately frozen.

### 4.2. Purification of the Peptides

Concentration and partial purification of the peptides in the pooled skin secretions was carried out using Sep-Pak C-18 cartridges (Waters Associates, Milford, MA, USA). Purification to near homogeneity was accomplished by means of successive chromatographies on semiprepatative (1.0 cm × 25 cm) Vydac 218TP510 (C-18), Vydac214TP510 (C-4) and Vydac 208TP51 (C-8) reversed-phase HPLC columns (Grace, Deerfield, IL, USA) as previously described [36]. Full details are provided as Supplementary Materials.

### 4.3. Structural Characterization

MALDI-TOF mass spectrometry was carried out using an UltrafleXtreme instrument (Bruker Daltonik, Bremen, Germany). Full details of the procedure, including calibration of the instrument with peptides of known molecular mass in the 1–4 kDa range, have been provided previously [37]. The accuracy of mass determinations was <0.02%. The primary structures of the purified peptides were determined via automated Edman degradation using an Applied Biosystems model 494 Procise sequenator (Applied Biosystems, Courtaboeuf, France).

### 4.4. Peptide Synthesis

Figainin 2BN, picturin 1BN and picturin 2BNwere supplied in crude form by PEPMIC (Suzhou, China) and were purified to near homogeneity (>98% purity) via reversed-phase HPLC on a (2.2 cm × 25 cm) Vydac 218TP1022 (C-18) column under the conditions previously described [36]. Full details are provided as Supplementary Materials. The identities of the peptides were confirmed through electrospray mass spectrometry.

### 4.5. Conformational Analysis

Secondary structure predictions were obtained using the Peptide Secondary Structure Prediction server, which predicts the secondary structure of peptides using a random forest classifier approach [19]. Circular Dichroism spectra were recorded on a MOS-500 Circular Dichroism Spectrometer (BioLogic, Seyssinet-Pariset, France) as previously described [38]. Full details are provided as Supplementary Materials. Figainin 2BN and picturin 1BN were dissolved in water at a concentration of 0.5 mg∙mL^−1^, and the solution was used to prepare samples containing TFE (25% and 50%), 20 mmol∙L^−1^ DPC and 10 mmol∙L^−1^ SDS at a 0.25 mg∙L^−1^ peptide concentration. Figainin 2BN was insoluble in 20 mmol∙L^−1^ SDS. Circular dichroism measurements are reported as mean residue molar ellipticity ([θ]MRE (deg∙cm^2^∙dmol^−1^)).

Peptide secondary structure was estimated using the online CD spectra deconvolution server Dichroweb [22,23,24]. The secondary structure content was determined through averaging the results obtained from CONTINLL [39,40], CDSSTR [41,42] and SELCON3 [43,44] deconvolution programs. Peptide α-helical content was also calculated using the Forood formula: 100 × ([θ]_222_/max[θ]_222_) with max[θ]_222_ = − 40,000 [1 − (2.5/n)], where n = number of amino acid residues [25].

### 4.6. Antimicrobial Assays

Reference strains of microorganisms were purchased from the American Type Culture Collection (Rockville, MD, USA). Minimum inhibitory concentration (MIC) of the purified peptides against *Escherichia coli* (ATCC 25922), *Klebsiella pneumoniae* (ATCC 49472), *Pseudomonas aeruginosa* (ATCC 9072), *Enterococcus faecium* (ATCC 19439), *Enterococcus faecalis* (ATCC 51299) and ampicillin-resistant *Staphylococcus aureus* (ATCC 12600) were determined in duplicate by means of a standard double dilution method according to Clinical Laboratory and Standards Institute (CSLI) guidelines [42] using 96-well microtiter cell-culture plates, as previously described [45]. Control incubations were carried out in parallel with increasing concentrations of vancomycin for *S. aureus*, ampicillin for *E. faecalis* and *E. coli* and ciprofloxacin for *K. pneumoniae* and *P. aeruginosa* in order to monitor the validity and reproducibility of the assays.

### 4.7. Cytotoxicity Assays

Cytotoxicity against human non-small cell lung adenocarcinoma A549 cells, human breast adenocarcinoma MDA-MB-231 cells, human colorectal adenocarcinoma HT-29 cells and human umbilical vein endothelial cells (HUVECs) were measured as previously described [29,46]. The effects of the peptides (1–100 μM) on cell viability were determined through measurement of ATP concentrations using a CellTiter-Glo Luminescent Cell Viability assay (Promega Corporation, Madison, WI, USA) following 24 h of incubation. The LC_50_ value was taken as the mean concentration of peptides producing 50% cell death in a minimum of three independent experiments.

## 5. Conclusions

The study has identified a peptide, figainin 2BN, present in norepinephrine-stimulated skin secretions from the frog *B. boans* that inhibits the growth of a range of clinically relevant Gram-negative and Gram-positive bacteria with relatively high potency (MIC ≤ 31.3 µM). Two additional peptides, picturin 1BN and picturin 2BN, were identified that were potently active against Gram-negative bacteria (MIC ≤ 15.6 µM). However, the possibility of their being developed into therapeutically valuable anti-infective agents, particularly for systemic use, is limited by strong cytotoxic activity against a range of human-derived cell lines. Future studies will address the design of analogs of these peptides containing appropriate amino acid substitutions that maintain, or increase, potencies against pathogenic microorganisms while reducing toxicities against mammalian cells. Strategies such as judicious modifications of cationicity (substitution of neutral and acidic residues with L-lysine), hydrophobicity (substitution of hydrophilic residues with L-leucine or L-tryptophan), conformation (incorporation of helix-stabilizing residues such as α-aminoisobutyric acid) and hydrophobic moment (substitution of L-lysine residues with D-lysine) are well established [47].

## Figures and Tables

**Table 1:** Primary structures and molecular masses of the peptides isolated from norepinephrine-stimulated skin secretions from *B. boans*.

Peptide	Primary Structure	[MH^+^]exp	[MH^+^]calc
Tryptophyllin BN	WRPFPFL.NH_2_	961.5	961.5
Tryptophyllin BN non-amidated	WRPFPFL	962.5	962.5
Figainin 2BN	FLGVALKLGKVLGKALLPLASSLLHSQ	2773.8	2773.7
[Q27E]figainin 2BN	FLGVALKLGKVLGKALLPLASSLLHSE	2774.8	2774.7
Picturin 1BN	GIFKDTLKKVVAAVLTTVADNIHPK	2678.7	2678.6
[N21D]picturin 1BN	GIFKDTLKKVVAAVLTTVADDIHPK	2679.6	2679.6
Picturin 2BN	GLMDMLKKVGKVALTVAKSALLP	2383.4	2383.4

[MH^+^]exp denotes the experimentally determined molecular mass and [MH^+^]calc denotes the mass calculated from the proposed structures. The values are expressed as Daltons.

**Table 2:** Physicochemical properties of the host-defense peptides isolated from norepinephrine-stimulated skin secretions from *B. boans*.

Peptide	Charge at pH 7.0	Hydrophobicity	Hydrophobic Moment	Predicted Helical Domain
Figainin 2BN	+3	0.667	0.348	4–25
[Q27E]figainin 2BN	+2	0.651	0.337	4–25
Picturin 1BN	+2	0.405	0.250	5–19
[N21D]picturin 1BN	+1	0.398	0.244	5–19
Picturin 2BN	+3	0.511	0.342	3–19

Mean hydrophobicity using the hydrophobicity scale of Fauchere and Pliska [16] and hydrophobic moment [17], a measure of the amphipathicity of an α-helix, were calculated using the HeliQuest web-server [18]. Predicted helical domains were calculated using the PEP2D program [19].

**Table 3:** Prediction of secondary structure content from CD spectra of figainin 2BN and picturin 1BN from *B. boans*. Values are given in %.

Peptide	Medium	Method	Helix	β Sheet	Turns	Random
Figainin 2BN	water	Dichroweb	2	21	10	33
		[θ]_222_	1	-	-	-
	25% TFE	Dichroweb	35	18	20	28
		[θ]_222_	27	-	-	-
	50% TFE	Dichroweb	40	17	17	27
		[θ]_222_	33	-	-	-
	20 mM DPC	Dichroweb	41	9	15	22
		[θ]_222_	42	-	-	-
	10 mM SDS	Dichroweb	49	9	14	26
		[θ]_222_	38			
Picturin 1BN	water	Dichroweb	2	4	2	89
		[θ]_222_	6	-	-	-
	25% TFE	Dichroweb	61	7	12	22
		[θ]_222_	58	-	-	-
	50% TFE	Dichroweb	64	3	11	24
		[θ]_222_	59	-	-	-
	20 mM DPC	Dichroweb	69	0	10	23
		[θ]_222_	61	-	-	-
	10 mM SDS	Dichroweb	65	3	9	22
		[θ]_222_	57	-	-	-

[θ]_222_ corresponds to the % helicity calculated using the Forood formula [25]. TFE—2,2,2-trifluoroethanol; DPC—dodecylphosphocholine; SDS—sodium dodecyl sulfate.

**Table 4:** Minimum inhibitory concentrations in μM of synthetic replicates of HDPs isolated from skin secretions of *B. boans* against reference strains of Gram-positive and Gram-negative bacteria.

Microrganism	Figainin 2BN	Picturin 1BN	Picturin 2BN
Gram-positive			
*S. aureus* (ATCC 12600)	7.8	62.5	31.3
*E. faecium* (ATCC 19439)	31.3	ND	ND
*E. faecalis* (ATCC 51299)	125	ND	ND
Gram-negative			
*E. coli* (ATCC 35218)	15.6	7.8	7.8
*K. pneumoniae* (ATCC 49472)	31.3	15.6	15.6
*P. aeruginosa* (ATCC 9072)	31.3	15.6	15.6

ND: not determined.

**Table 5:** Cytotoxicities of peptides from *B. boans* against lung adenocarcinoma A549 cells, breast adenocarcinoma MDA-MB-231 cells, colorectal adenocarcinoma HT-29 cells and human umbilical vein endothelial cells (HUVECs).

Cells	Figainin 2BN	Picturin 1BN	Picturin 2BN
A549	6.7 ± 0.4	30.7 ± 0.6	23.0 ± 0.7
MDA-MB-231	8.1 ± 0.5	64.2 ± 0.9	26.3 ± 1.9
HT-29	14.1 ± 0.8	84.9 ± 4.3	52.2 ± 3.1
HUVEC	15.1 ± 0.5	54.0 ± 1.2	53.3 ± 2.4

Data show mean LC_50_ values (μM) ± S.E.M.

## Data Availability

The data presented in this study are available on request from the corresponding author.

## Supplementary Materials

The following supporting information can be downloaded at: https://www.mdpi.com/article/10.3390/antibiotics12071102/s1.
