# Supplementary material for: Purification, Conformational Analysis and Cytotoxic Activities of Host-Defense Peptides from the Giant Gladiator Treefrog Boana boans (Hylidae: Hylinae)
Source: Antibiotics (Basel). 2023 Jun 25;12(7):1102. doi: 10.3390/antibiotics12071102 (PMC10376367; doi:10.3390/antibiotics12071102)
Supplement: Supplementary file 1 [file antibiotics-12-01102-s001.zip › antibiotics-2448978-supplementary-done.pdf]

<u>Supplementary material</u>

Purification, Conformational Analysis and Cytotoxic Activities of Host-Defense Peptides from the Giant Gladiator Treefrog *Boana boans* (Hylidae: Hylinae)

J. Michael Conlon, Laure Guilhaudis, Samir Attoub, Laurent Coquet, Jérôme Leprince, Thierry Jouenne and Milena Mechkarska

Purification of the peptides

The solutions containing the secretions were pooled and partial purification was carried out by passage at a flow rate of approximately 2 mL.min^{-1} through 6 Sep-Pak C-18 cartridges (Waters Associates, Milford, MA, USA) connected in series. Bound material was eluted with acetonitrile/water/trifluoroacetic acid (TFA) (70.0:29.9:0.1, v/v/v) and freeze-dried. The material was redissolved in 0.1% (v/v) TFA/water (2 mL) and injected onto a semipreparative (1.0 cm x 25 cm) Vydac 218TP510 (C-18) reversed-phase HPLC column (Grace, Deerfield, IL, USA) equilibrated with 0.1% (v/v) TFA/water at a flow rate of 2.0 mL.min^{-1}. The concentration of acetonitrile in the eluting solvent was raised to 21% (v/v) over 10 min and to 63% (v/v) over 60 min using linear gradients. Absorbance was monitored at 214 nm and fractions (1 min) were collected using a BioRad 2110 fraction collector. The peptides within the peaks designated 1 - 8 (Figure 1) that were present in major abundance were subjected to further purification by successive chromatographies on (1.0 cm x 25 cm) Vydac 214TP510 (C-4) and (1.0 cm x 25 cm) Vydac 208TP510 (C-8) columns. The concentration of acetonitrile in the eluting solvent was raised from 21% to 56% (v/v) over 50 min for peaks 1 - 3 and from 28% to 63% over 50 min for peaks 4 - 8. The flow rate was 2.0 mL.min^{-1} and fractions were collected by hand.

Peptide synthesis

Figainin 2BN, picturin 1BN and picturin 2BNwere supplied in crude form by PEPMIC (Suzhou, China) and were purified to near homogeneity (> 98% purity) by reversed-phase HPLC on a (2.2 cm x 25 cm) Vydac 218TP1022 (C-18) column equilibrated with acetonitrile/water/ TFA (35.0/64.9.9.9/0.1, v/v/v) at a flow rate of 6 mL.min^{-1}. The concentration of acetonitrile was raised to 63% (v/v) over 60 min using a linear gradient. Absorbance was measured at 214 nm and the major peak in the chromatogram was collected manually. The identities of the peptides were confirmed by electrospray mass spectrometry.

Conformational analysis

Circular Dichroism spectra were recorded on a MOS-500 Circular Dichroism Spectrometer (BioLogic, Seyssinet-Pariset, France). Data

points were collected from 260 to 185 nm, with an integration time of 2 s per point and a step size of 1 nm, using a 1.0 mm path length rectangular quartz cell. Measurements were carried out at room temperature. Figainin 2BN and picturin 1BN were dissolved in water at a concentration of 0.5 $mg.mL^{-1}$ and the solution was used to prepare samples containing TFE (25% and 50%), 20 $mmol.L^{-1}$ DPC and 10 $mmol.L^{-1}$ SDS at a 0.25 $mg.L^{-1}$ peptide concentration. Figainin 2BN was insoluble in 20 $mmol.L^{-1}$ SDS. Three scans were accumulated and averaged for each sample. All spectra were corrected by subtraction of the background obtained for each peptide-free solution. Circular dichroism measurements are reported as mean residue molar ellipticity ($[\theta]$MRE ($deg.cm^2.\ dmol^{-1}$).
